# Summary of the best evidence for the prevention of deep vein thrombosis in patients with intracerebral hemorrhage

**DOI:** 10.3389/fneur.2026.1752010

**Published:** 2026-01-27

**Authors:** Wenguang Xie, Qingxin Xu, Yangyang Zhang, Yanyan Gong, Wei Xiao, Wenhao Zhang, Shuyuan Huang, Huan Li, Chao Zhang

**Affiliations:** 1 The 2nd Affiliated Hospital, Jiangxi Medical College, Nanchang University, Nanchang, China; 2 School of Nursing, Jiangxi Medical College, Nanchang University, Nanchang, China; 3 Department of Songbei General Surgery, The Fourth Affiliated Hospital of Harbin Medical University, Harbin, China; 4 Department of Nursing, The First Affiliated Hospital of University of Science and Technology of China (Anhui Provincial Hospital), Hefei, China; 5 Neurosurgery ICU, The Second Affiliated Hospital of Nanchang University, Nanchang, China; 6 Department of Neurosurgery, The Second Affiliated Hospital of Nanchang University, Nanchang, China

**Keywords:** cerebral hemorrhage, deep vein thrombosis, evidence summary, evidence-based nursing, prevention

## Abstract

**Objective:**

This study aims to retrieve, evaluate, and summarize the existing evidence regarding the prevention of deep vein thrombosis (DVT) in patients with cerebral hemorrhage. The findings will provide a solid foundation for clinical nursing practice.

**Design:**

This study presents a comprehensive evidence summary conducted in accordance with the standards set forth by the Evidence-Based Nursing Center at Fudan University. The adherence to these standards ensures the rigor and reliability of the findings presented herein.

**Methods:**

Based on the ‘5S’ evidence pyramid model, various evidence-based resources for the prevention of deep vein thrombosis in patients with cerebral hemorrhage were systematically retrieved. These resources include clinical decisions, best practices, guidelines, expert consensus, systematic reviews, and evidence summaries. The search period spans from January 2011 to April 2025.

**Results:**

This study included a total of 16 high-quality articles, comprising 2 clinical decisions, 7 guidelines, 4 expert consensuses, 2 systematic reviews, and 1 summary of evidence. In total, 38 pieces of evidence were synthesized across six dimensions: risk assessment, basic prevention, mechanical prevention, pharmacological prevention, nursing documentation, and informed consent.

**Conclusion:**

This study summarizes the 38 most compelling pieces of evidence for the prevention of DVT in patients with cerebral hemorrhage, providing an evidence-based foundation for clinical medical staff. It is recommended that healthcare professionals implement these evidence-based practices in clinical settings to effectively reduce the incidence of DVT among patients suffering from cerebral hemorrhage.

**Systematic review registration:**

http://ebn.nursing.fudan.edu.cn/registerResources, identifier ES2025786.

## 1 Introduction

Intracerebral hemorrhage (ICH) refers to brain injury caused by the non-traumatic rupture of blood vessels in the brain, resulting in blood seeping from the ruptured cerebral vessels into the brain parenchyma. It is a subtype of stroke, with an incidence rate second only to that of ischemic stroke (1). According to a study by the World Stroke Organization, stroke was the third leading cause of death globally in 2021, accounting for 28.8% of all stroke patients with cerebral hemorrhage (2). China faces the world’s largest challenge concerning stroke. Data from the China Hospital Quality Monitoring System and the China Stroke Big Data Observation Platform indicate that in 2020, there were a total of 3,418,432 stroke patients in China, of which 14.9% were patients with cerebral hemorrhage. The hospitalization medical expenses for stroke patients reached as high as 58 billion yuan, imposing a significant economic burden (3). ICH is the most fatal subtype of acute stroke, with a mortality rate of patients within 3 months of illness reaching as high as 30 to 40%. Only about 20% of patients can restore their life and self-care ability after 6 months, resulting in a substantial disease burden for society and families (1, 4). Patients with cerebral hemorrhage often require admission to the neurosurgical ICU following the onset of the disease. During the neurointensive care period, 26.97% of these patients may develop in-hospital complications, which significantly increases the mortality rate and prolongs hospital stays (5). Among these complications, deep vein thrombosis is one of the most common in patients with cerebral hemorrhage.

Deep vein thrombosis (DVT) is a disorder characterized by impaired venous return due to abnormal blood coagulation in the deep veins. It predominantly affects the lower extremities and is a condition that can be effectively prevented (6). DVT serves as the primary source of pulmonary embolism. In the acute phase, the detachment of emboli can result in a sudden mortality rate of up to 30% among patients, with a one-year mortality rate of 20% for those who survive following diagnosis (6, 7). DVT is also known for its propensity to recur, with approximately 30% of patients experiencing a recurrence within 10 years of their initial diagnosis (8). Additionally, a significant chronic complication of DVT is post-thrombotic syndrome, which affects approximately 20 to 50% of patients. This syndrome can lead to anxiety, depression, and a decreased quality of life, imposing a substantial economic burden on both patients and their families (7, 8). Patients with cerebral hemorrhage are at a heightened risk of developing DVT due to several factors, including consciousness disorders, hemiplegia, prolonged hospital stays, intubation, intravascular hemorrhage, a hypercoagulable state, and inadequate early pharmacological prevention (1, 9, 10). Studies indicate that the incidence of DVT detected by ultrasound in patients with cerebral hemorrhage admitted to the neurosurgical intensive care unit can reach as high as 20 to 40%, which is four times greater than that observed in cases of acute ischemic cerebral hemorrhage (1, 9). In China, study reported that the incidence of VTE in ICH patients during hospitalization ranges from 8.56 to 48.9%, with pulmonary embolism occurring in approximately 2.9% of cases (11, 12). These figures underscore the urgent need for effective preventive strategies in this high-risk population. Therefore, the prevention of DVT during the neurointensive care of patients with cerebral hemorrhage is of paramount importance.

In China, the primary prevention methods for DVT in patients with cerebral hemorrhage include mechanical and pharmacological prophylaxis, which predominantly rely on clinical experience and traditional practices, thereby lacking a comprehensive evidence-based summary (13). Research indicates a significant disparity between the current practices of DVT prevention in Chinese patients with cerebral hemorrhage and established guidelines. Specifically, only 14.2% of patients initially received treatment in a neurointensive care unit, and merely 22.3% received preventive treatment for DVT within 48 h of onset (14). Furthermore, the implementation rate for early DVT prevention stands at 49.9%, while the rate for early mobilization is only 29.49%, with pharmacological prevention being a mere 2.02%. These figures indicate a substantial gap in adherence to guidelines (15). This discrepancy is primarily due to the absence of a consolidated evidence summary and the lack of evidence-based practice plans for DVT prevention in this patient population in China. Additionally, recent years have seen a proliferation of relevant guidelines concerning DVT prevention in patients with cerebral hemorrhage (1, 16–18), with continuous emergence of new evidence and revisions of existing data. To facilitate the translation of evidence into clinical practice, this study aims to compile the best available evidence for DVT prevention in patients with cerebral hemorrhage, thereby creating a scientific and effective evidence-based resource. This summary is anticipated to assist clinical medical personnel in standardizing their practices, reducing DVT incidence in patients, ensuring patient safety, and providing a foundation for the development of an evidence-based practice plan for DVT prevention in patients with cerebral hemorrhage, as well as guiding future scientific research on its implementation.

## 2 Methods

Currently, there is a notable absence of standardized reporting norms for evidence summaries. This study is grounded in the reporting standards developed by the Evidence-Based Nursing Center of Fudan University, which are based on evidence generated by the Joanna Briggs Institute (JBI) (19). Detailed methodological descriptions, including search strategies, inclusion/exclusion criteria, quality appraisal tools, and evidence grading process, are provided in Supplementary material S1.

## 3 Results

### 3.1 Literature retrieval results

**Figure 1:**
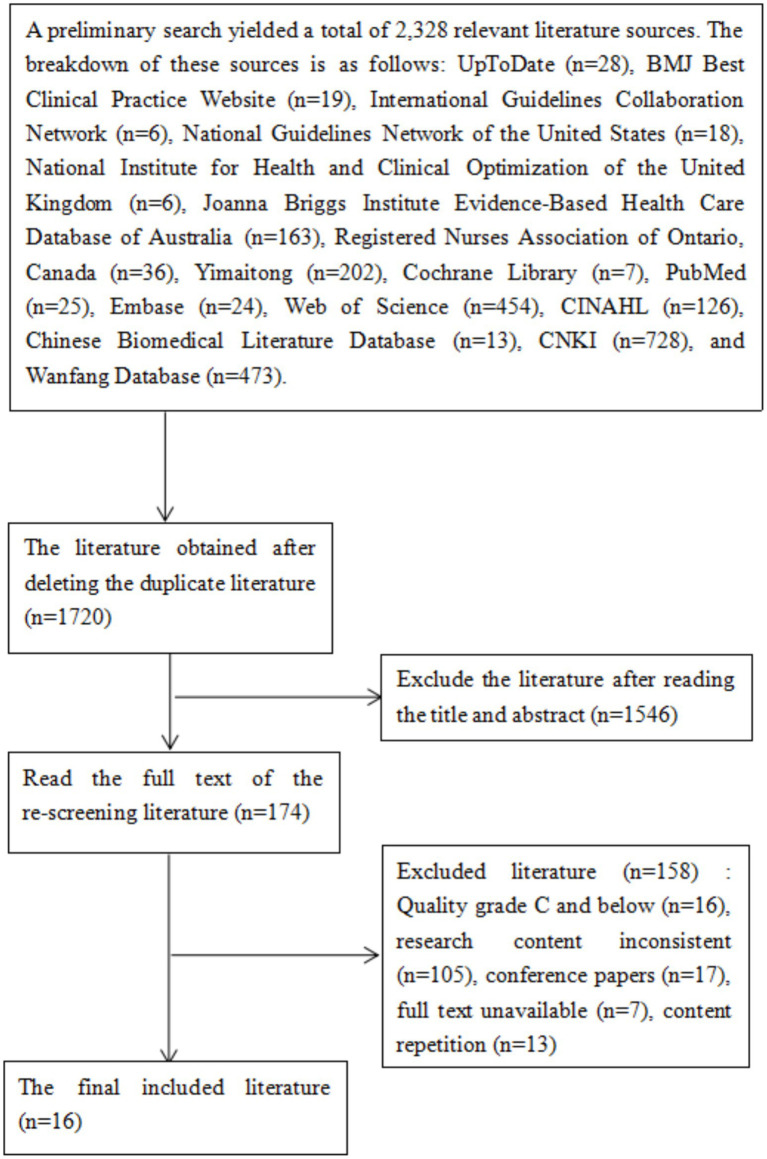
Flow chart of literature screening.

A total of 2,328 literature sources were obtained through the initial search for this study. After importing these sources into the literature management software EndNote 20, 608 duplicates were removed. Consequently, 1,720 literature sources remained. Two researchers independently reviewed the titles and abstracts of the remaining sources and initially excluded 1,546 of them. Following a thorough examination of the full texts, 158 sources that did not meet the inclusion criteria for this study were further excluded. Ultimately, 16 sources were included in this study, comprising 2 related to clinical decision-making (20, 21), 7 pertaining to guidelines (1, 16–18, 22, 23), 2 systematic reviews (24, 25), 4 expert consensus documents (26–29), and 1 evidence summary (30). The literature screening process is illustrated in Figure 1, and the general characteristics of the included literature are summarized in Table 1.

**Table 1:** General characteristics of the included literature (*n* = 16).

Included literature	Publication time (year)	Country	Source of literature	Literature topic	Literature type
Ishida (20)	2024	The United States	UpToDate	Prevention and treatment of venous thromboembolism in patients with acute stroke	Clinical decision
Rordorf et al. (21)	2025	The United States	UpToDate	Spontaneous intracerebral hemorrhage: Acute treatment and prognosis	Clinical decision
NICE (22)	2018	Britain	NICE	reducing the risk of hospital-acquired deep vein thrombosis or pulmonary embolism	Guideline
Greenberg et al. (1)	2022	The United States	PubMed	2022 Guideline for the management of patients with spontaneous intracerebral hemorrhage: a guideline from the American Heart Association/American Stroke Association	Guideline
Mora et al. (16)	2024	Spain	Web of Science	European guidelines on peri-operative venous thromboembolism prophylaxis: first update. Chapter 6: neurosurgery	Guideline
Steiner et al. (23)	2014	Germany	Web of Science	European Stroke Organisation (ESO) guidelines for the management of spontaneous intracerebral hemorrhage	Guideline
Nyquist et al. (9)	2016	The United States	PubMed	Prophylaxis of Venous Thrombosis in Neurocritical Care Patients: An Evidence-Based Guideline: A Statement for Healthcare Professionals from the Neurocritical Care Society	Guideline
Qian et al. (17)	2023	China	CNKI	Chinese Stroke Association Guidelines for Clinical Management of Cerebrovascular Diseases—Chapter Five Clinical Management of Intracerebral Hemorrhage	Guideline
Neurology Branch of the Chinese Medical Association, et al. (18)	2024	China	Wanfang Database	Chinese guidelines for the management of severe stroke 2024	Guideline
Diao et al. (24)	2024	China	PubMed	Risk factors and predictors of venous thromboembolism in patients with acute spontaneous intracerebral hemorrhage: A systematic review and meta-analysis	systematic review
Chi et al. (25)	2022	The United States	PubMed	Systematic Review and Meta-Analysis of Thromboprophylaxis with Heparins Following Intracerebral Hemorrhage	systematic review
Shanghai Pulmonary Embolism and Deep Vein Thrombosis Prevention and Treatment Alliance et al. (26)	2022	China	Wanfang Database	China expert consensus on intermittent intermittent pneumatic compression for prevention of venous thromboembolismy	Expert consensus
Expert Committee of the Thrombosis and Vascular Special Fund of the China Health Promotion Foundation (27)	2020	China	Yimaitong	Chinese expert consensus on mechanical prevention of venous thromboembolism	Expert consensus
Yan et al. (28)	2019	China	CNKI	Expert consensus on nursing standards for subcutaneous injection of anticoagulant agents	Expert consensus
Nursing Professional Committee of the China Branch of the International Vascular Union (29)	2021	China	Yimaitong	Expert consensus on preventive nursing and management of venous thromboembolism in inpatients	Expert consensus
Ting et al. (30)	2019	China	CNKI	Evidence summary for mechanical prophylaxis of venous thromboembolism in patients with intracerebral hemorrhage	Summary of evidence

### 3.2 Literature quality evaluation results

#### 3.2.1 The quality evaluation results of clinical decision-making and evidence summary

This study encompasses two clinical decisions (20, 21) and one evidence summary (30). A CASE list was utilized to assess the quality of the literature (31). The evaluation results indicate that the quality of all three sources is exceptionally high, warranting their inclusion in this study. Detailed information is presented in Table 2.

**Table 2:** Literature quality evaluation results of clinical decision-making and evidence summary (*n* = 3).

Items	Ishida (20)	Rordorf et al. (21)	Ting et al. (30)
The scope and objects are specific	Yes	Yes	Yes
The author is clear and transparent.	Yes	Yes	Yes
The review is clear and transparent.	Yes	Yes	Yes
The retrieval is transparent and comprehensive	Yes	Yes	Yes
The classification of evidence is clear.	Yes	Yes	Yes
The recommendation opinion is clear.	Yes	Yes	Yes
The recommendation is appropriately cited	Yes	Yes	Yes
Recommendation opinions have a time limit	Yes	Yes	Yes
Statement of Conflict of Interest	Yes	Yes	No
Applicable to the population of this study	Yes	Yes	Yes

#### 3.2.2 Quality evaluation results of guidelines

This study encompasses a total of seven guidelines (1, 9, 16–18, 22, 23), of which two originate from the United States (1, 9), two from China (17, 18), one from Germany (23), one from Spain (16), and one from the United Kingdom (22). Three researchers independently assessed the quality of the literature pertaining to these guidelines using the AGREE II tool. Based on the standardized percentage scores across various domains, six guidelines received a rating of Grade A, while one guideline was rated as Grade B. Detailed information is presented in Table 3.

**Table 3:** Literature quality evaluation results of the guidelines (*n* = 7).

Included literature	Percentage of standardization in each field of the guideline (%)						≥60% Number of fields (pieces)	≥30% number of fields (pieces)	Recommendation level
	Scope and purpose	Stakeholder involvement	Rigor of development	Clarity of presentation	Applicability	Editorial independence			
NICE (22)	100	75.00	90.48	87.50	70.37	97.22	6	6	A
Greenberg et al. (1)	100	77.78	98.41	97.22	83.33	100	6	6	A
Mora et al. (16)	96.30	61.11	90.48	84.72	77.78	88.89	6	6	A
Steiner et al. (23)	90.74	45.83	78.57	83.33	53.70	91.67	4	6	B
Nyquist et al. (9)	100	68.05	88.10	90.28	70.37	88.89	6	6	A
Qian et al. (17)	96.30	62.50	100	90.28	74.07	100	6	6	A
Neurology Branch of the Chinese Medical Association et al. (18)	90.74	61.11	60.32	86.11	74.07	100	6	6	A

#### 3.2.3 Quality evaluation results of systematic reviews

This study encompasses two systematic reviews (24, 25), one conducted in China (24) and the other in the United States (25). Both reviews were published within the last 5 years and focus on the prevention of DVT in patients with cerebral hemorrhage. Following a thorough evaluation of the literature quality, all items in both systematic reviews received a positive assessment, indicating a high level of quality. Consequently, both reviews were included in this study. Detailed information is presented in Table 4.

**Table 4:** Literature quality evaluation results of the systematic review (*n* = 2).

Items	Evaluation results	
	Diao et al. (24)	Chi et al. (25)
Are the evidence-based questions raised clear and definite?	Yes	Yes
2. Are the inclusion criteria of the literature appropriate for this evidence-based issue?	Yes	Yes
3. Is the retrieval strategy appropriate?	Yes	Yes
4. Are the databases or resources for retrieving literature adequate?	Yes	Yes
5. Are the adopted criteria for evaluating the quality of literature appropriate?	Yes	Yes
6. Was the literature quality evaluation independently completed by two or more reviewers?	Yes	Yes
7. Were certain measures taken when extracting data to reduce errors?	Yes	Yes
8. Is the method of combined research appropriate?	Yes	Yes
9. Has the possibility of publication bias been evaluated?	Yes	Yes
10. Are the policy or practice recommendations put forward based on the results of systematic evaluation?	Yes	Yes
11. Are the proposed further research directions appropriate?	Yes	Yes

#### 3.2.4 Quality evaluation results of expert consensus

This study encompasses a total of four expert consensuses (26–29), all originating from China. In the third expert consensus (28), with the exception of the second entry, “Does the author have a certain influence in this field?,” which received an evaluation result of “Unclear,” all other evaluation results were “Yes.” Similarly, in the fourth expert consensus (29), apart from the sixth item, “Are there any inconsistencies between the viewpoints proposed and previous literature?,” which also resulted in an evaluation of “Unclear,” all other items received a “Yes” evaluation. Furthermore, the evaluation results for all items in the remaining two expert consensus papers (26, 27) were uniformly “Yes.” The overall literature quality evaluation results of the four expert consensuses were notably high, warranting their inclusion in this study. Detailed information is presented in Table 5.

**Table 5:** Literature quality evaluation results of expert consensus (n = 4).

Items	Evaluation results			
	Shanghai Pulmonary Embolism and Deep Vein Thrombosis Prevention and Treatment Alliance et al. (26)	Expert Committee of the Thrombosis and Vascular Special Fund of the China Health Promotion Foundation (27)	Yan et al. (28)	Nursing Professional Committee of the China Branch of the International Vascular Union (29)
1. Is the source of the viewpoint clearly marked?	Yes	Yes	Yes	Yes
2. Does the author have a certain influence in this field?	Yes	Yes	Unclear	Yes
3. Are the viewpoints proposed centered on studying the interests of the relevant population?	Yes	Yes	Yes	Yes
4. Are the viewpoints stated based on the results of the analysis and is the expression of the viewpoints logical?	Yes	Yes	Yes	Yes
5. Has any existing literature been referred to and accurately indexed?	Yes	Yes	Yes	Yes
6. Are there any inconsistencies between the viewpoints proposed and those in previous literature?	Yes	Yes	Yes	Unclear

### 3.3 Summary and description of evidence

Following the description and summarization of the evidence presented in this study, nine experts were convened for an expert group meeting. This group comprised one specialist in neurocritical care medicine, one in vascular surgery medicine, two in neurocritical care nursing, one in vascular surgery nursing, one in ultrasound medicine, one in rehabilitation medicine, one in evidence-based nursing, and one in nursing management. The extracted and summarized evidence was evaluated according to the FAME attributes, and the strength of the recommendations was assessed based on the principles outlined in the JBI Evidence Pre-Grading and Evidence Recommendation Level System (2014 Edition) (32). Ultimately, this study identified 38 pieces of best evidence across six domains: risk assessment, basic prevention, mechanical prevention, pharmacological prevention, nursing documentation, and informed consent. For further details, please refer to Table 6.

**Table 6:** Summary of the best evidence for the prevention of deep vein thrombosis in patients with cerebral hemorrhage.

Category	Content of evidence	Level	Main implementer	Recommendation level
Risk assessment	1. Medical staff should attach importance to the prevention of DVT in patients with cerebral hemorrhage, and take individualized prevention and treatment measures after assessing the risks and weighing the benefits (18)	3	N/D	A
	2. Medical staff should complete the DVT risk assessment and hemorrhage risk assessment within 24 h after the admission of patients with cerebral hemorrhage, and within 6 h after the operation and transfer to another department. They should reevaluate before the patient is discharged and conduct dynamic re-evaluation when the patient’s condition changes (29, 30)	5	N/D	A
	3. Medical staff should use the Caprini assessment form to assess the thrombosis risk of patients with cerebral hemorrhage (29)	5	N/D	A
	4. Before deciding whether to use anticoagulant drugs for thrombosis prevention, medical staff should assess the patient’s bleeding risk (22)	1	N/D	A
	5. Medical staff should conduct bleeding risk assessment for patients with cerebral hemorrhage based on the related factors of bleeding risk. Regular assessment should be carried out during the period when patients change anticoagulant drugs again, their condition worsens, undergo surgery, and apply anticoagulant drugs (29)	5	N/D	A
	6. Hemiplegia of limbs, advanced age, previous history of DVT, obesity, malignant tumors, infections, intubation, intraventricular hemorrhage, long operation time and prolonged hospital stay are high-risk factors for DVT in patients with cerebral hemorrhage, and assessment should be strengthened (16, 20, 24, 27)	2	N/D	A
	7. Nurses should correctly measure the circumference of the limbs at least once a day and accurately assess the degree of limb swelling (30)	5	N	A
	8. For patients at high risk or suspected of having DVT, D-dimer and limb venous Doppler ultrasound examinations can be performed (18)	3	D	B
Basic prevention	9. For conscious patients with stable conditions, nurses should encourage them to engage in early activities and leg exercises (18, 22, 29)	1	N	A
	10. For conscious patients, nurses should guide them to perform ankle pump exercises. For patients with poor compliance, nurses should assist them in performing passive ankle pump exercises (29)	5	N	B
	11. When there are no contraindications for the patient, the nurse should elevate the patient’s lower limbs (18)	3	N	A
	12. When the patient’s condition permits, the nurse should, as per the doctor’s advice, provide the patient with appropriate fluid replacement to prevent blood concentration (22)	1	N	B
Mechanical prevention	13. For immobile patients with cerebral hemorrhage, we recommend using IPC to prevent DVT starting from the first day of admission (1, 17, 20, 21, 23)	1	N/D	A
	14. When using IPC, it is recommended to apply pressure to the thighs and/or calves. The appropriate pressure range is 35 to 40 mmHg. Inflate the leg brace for about 10 s each time, then relax for 1 min, and repeat the process (26)	5	N	A
	15. For patients with cerebral hemorrhage, it is recommended to use IPC for no less than 18 h per day for 30 days or until the patient resumes normal activities or is discharged from the hospital, whichever is earlier (22, 30)	1	N/D	B
	16. When using IPC, it is necessary to avoid direct contact of the leg covers with the patient’s lower limb skin. It is recommended to wrap the leg covers around the outer layer of thin and smooth patient pants (26)	5	N	B
	17. It is recommended that nurses check the position of the leg covers, the pressure and the skin integrity and cleanliness of the pressurized areas at least once a day (26, 30)	5	N	A
	18. For leg covers that patients reuse, nurses should wipe and disinfect the surface with disinfectants such as 75% alcohol to prevent cross-infection (26)	5	N	A
	19. When the number of IPCs in the department cannot meet the needs of patients, it is recommended to adopt other mechanical preventive measures as substitutes (26)	5	N	A
	20. The contraindications for IPC include: obvious lower extremity ischemia caused by peripheral vascular disease, lower extremity ulcers, dermatitis, severe lower extremity edema, thrombophlebitis, severe limb deformity that makes it impossible to use compression sleeves, severe allergy to compression sleeves, congestive heart failure, and suspected or confirmed presence of DVT. Patients who have not received DVT prevention after cerebral hemorrhage and have been bedridden or immobilized for more than 72 h should not use IPC (20, 26, 27, 30)	5	N/D	A
	21. For patients with cerebral hemorrhage who are immobile, wearing knee-high or thigh stretch compression stockings alone cannot reduce the risk of DVT; instead, it increases the risk of skin damage. Therefore, it is not recommended (1, 17, 18, 20, 22, 23)	1	N/D	A
	22. It is recommended that nurses assess the compliance of patients before using mechanical devices (30)	1	N	A
	23. It is recommended that nurses formulate appropriate mechanical preventive care plans based on clinical situations, patients’ consciousness states and preferences, etc. (30)	3	N	A
Drug prevention	24. For immobile patients with cerebral hemorrhage, when the hematoma is confirmed to be stable 24–48 h after the onset, the use of preventive doses of unfractionated heparin or low-molecular-weight heparin can effectively prevent the occurrence of DVT in patients without increasing the risk of hematoma expansion or hemorrhage (1, 9, 16, 17, 20, 21, 25)	2	D	B
	25. For patients with cerebral hemorrhage who are immobile, it is recommended to combine mechanical prevention with drug prevention methods, which can maximize the preventive effect of DVT (9, 16, 27)	2	N/D	A
	26. The preferred injection site for anticoagulants is the abdominal wall, and the injection site should be changed regularly. The injection site on the abdominal wall is 1 cm below the left and right costal margins, 1 cm above the pubic symphysis, and 10 cm around the umbilicus, avoiding within 2 cm around the umbilicus (28, 29)	5	N	A
	27. For patients who need long-term subcutaneous injection of low-molecular-weight heparin, it is recommended that nurses use abdominal wall positioning cards for positioning before injection (28)	5	N	A
	28. During the operation of anticoagulant injection, the nurse needs to keep a distance of 5 to 6 cm between the thumb and the index finger of the left hand, pinch the patient’s skin to form a fold, and hold the syringe with the right hand in a pen-holding manner to puncture vertically at the top of the fold (28)	5	N	A
	29. It is recommended to use pre-filled anticoagulant injections. Before injection, no air should be vented, with the needle tip facing down. The air in the syringe should be gently flicked above the liquid medicine. After the needle is inserted, blood does not need to be drawn back before the liquid medicine is pushed in. After the liquid medicine is slowly pushed in, it should be left for 10 s before the needle is quickly removed (28, 29)	5	N	A
	30. After needle removal, there is no need to press. If there is bleeding or exudation at the puncture site, press vertically downward with the puncture point as the center for 3 to 5 min (28)	5	N	A
	31. Hot compress and physical therapy are contraindicated at the injection site after anticoagulant injection (28)	5	N	A
	32. During the medication period, nurses should dynamically observe the medication effect and whether there are any side effects such as bleeding. Once they occur, they should report to the doctor immediately (29)	5	N	A
Records of nursing documents	33. Nurses should accurately record in the nursing documents the risk factors of DVT in patients, the score of the Caprini assessment form and the thrombus prevention measures taken (29)	5	N	A
	34. During the use of IPC, nurses should promptly record relevant nursing documents, including the duration of compression, start and end times, adverse reactions of patients and measures taken, and the assessment of patients’ skin conditions, etc. (26, 29)	5	N	A
	35. During the period of drug prevention, nurses should accurately record the name, dosage, time, route of administration and adverse reactions of anticoagulant drugs in the nursing documents (29)	5	N	A
	36. During the prevention process of DVT in patients, nurses should truthfully record the thrombotic prevention measures taken and the reasons for the inconsistency with the prevention norms (29)	5	N	A
Informed consent	37. Before using IPC, medical staff should explain to patients with cerebral hemorrhage or their families the necessity of using IPC and the risks of adverse reactions such as skin injury and pulmonary embolism, and have the patients or their families sign the informed consent form (22, 26, 27)	3	N/D	A
	38. During the use of anticoagulants, medical staff should inform patients or their families of the necessity and potential risks of anticoagulant therapy, and require patients or their families to sign the “Informed Consent Form for Anticoagulant Therapy” after obtaining their informed consent (28)	5	N/D	A

N, Nurse-led; Doctor-led; N/D, The doctor and the nurse carried out the task together.

## 4 Discussion

### 4.1 The summary and formation process of this evidence is scientific and standardized, and has good clinical guiding significance

This study systematically retrieved evidence from top to bottom based on the 5S Evidence Golden Character Model (33). Following a thorough screening of the literature according to predefined inclusion and exclusion criteria, and a quality evaluation conducted by three researchers, 16 studies were ultimately included. These consist of 2 clinical decisions (20, 21), 7 guidelines (1, 16–18, 22, 23), 4 expert consensuses (26–29), 2 systematic reviews (24, 25), and 1 evidence summary (30). The included studies originate from five countries: China, the United States, Germany, Spain, and the United Kingdom. The AGREE II tool (34) was employed to assess the quality of the included guidelines. The results indicated that the quality of 7 guidelines was relatively high, suggesting that the guideline formulation process was rigorous, the methods were standardized, and the guidelines exhibited good applicability. The CASE list (35) was utilized to evaluate the quality of the clinical decision-making articles and the evidence summary. The findings revealed that both clinical decision-making articles and the evidence summary were of high quality. The two systematic reviews were assessed using the JBI Systematic Review and Integrated Research Assessment tool (36), yielding high-quality literature closely related to the prevention of DVT in patients with cerebral hemorrhage, characterized by a comprehensive research design. The four expert consensuses were evaluated using the quality assessment criteria of the JBI expert consensus (32), with all demonstrating relatively high quality. All literature included in this study was published within the last 10 years, ensuring the timeliness of the evidence. The process of literature quality evaluation was conducted independently by four researchers, ensuring rigor and reliability. Two researchers summarized the contents of the included evidence and extracted and organized the evidence by topic. Following the extraction, the research team utilized the JBI evidence pre-grading system to assess the evidence and determine the recommendation level based on the FAME structure of the JBI evidence, facilitated by an expert meeting with nine relevant experts (6). Consequently, the evidence formation process in this study is scientifically rigorous and can provide valuable guidance for clinical practice.

### 4.2 Risk assessment of DVT prevention in patients with cerebral hemorrhage

Risk assessment is the initial step in preventing DVT in patients with cerebral hemorrhage. This process primarily involves evaluating the risk of DVT, the potential for bleeding, and identifying high-risk factors associated with DVT in these patients. Current expert consensus (29) recommends utilizing the Caprini assessment tool for risk evaluation in this population. However, this instrument has limitations, including inadequate specificity and discrimination. Therefore, there is a need for the development of tailored risk assessment tools specifically for DVT occurrence in patients with cerebral hemorrhage. Nurses should utilize appropriate tools to assess both DVT and bleeding risks within the designated timeframe while being knowledgeable about the high-risk factors for DVT in this patient group. Currently, there is a need to improve nurses’ knowledge and attitudes regarding thrombosis prevention. Nursing managers should enhance training programs to elevate nurses’ understanding and attitudes towards DVT prevention (37, 38).

### 4.3 Drug prevention of DVT in patients with cerebral hemorrhage

Among patients with cerebral hemorrhage, the risk of cerebral hemorrhage associated with anticoagulant drugs often leads to early contraindications for drug prophylaxis (9). Several guidelines (1, 9, 16) indicate that for immobile patients with cerebral hemorrhage, when the hematoma is confirmed to be stable within 24–48 h post-onset, the use of preventive doses of unfractionated heparin or low-molecular-weight heparin can effectively prevent the occurrence of DVT without increasing the risk of hematoma expansion or hemorrhage. This provides robust evidence for clinical practice. Research conducted by the Chinese Stroke Center Alliance (15) reveals that the clinical implementation rate of DVT drug prevention in patients with cerebral hemorrhage is merely 2.02%, highlighting a significant gap between current practices and established guidelines. Future research should aim to bridge this gap between evidence and practice. During anticoagulant therapy, nurses must adhere strictly to operational procedures and standards, closely monitoring the therapeutic effects of anticoagulants and any adverse reactions, such as bleeding. Studies indicate that patients may experience adverse reactions, including pain and subcutaneous bruising, during anticoagulant injections administered by nurses. Nursing managers should establish standardized protocols for anticoagulant use, enhance nurse education and training, and improve nursing quality control to elevate the overall standard of care (39). However, duration of pharmacological prophylaxis** is another critical aspect of clinical decision-making. Current guidelines do not specify a uniform duration for anticoagulant use in ICH patients, as it should be individualized based on ongoing bleeding risk, mobility recovery, and hematoma stability. Future studies should aim to establish evidence-based recommendations on the optimal duration of thromboprophylaxis in this population.

### 4.4 Non-pharmacological prevention of DVT in patients with cerebral hemorrhage

In terms of basic prevention, nurses should encourage conscious patients to engage in early leg activities and perform leg exercises. Concurrently, they should guide patients in executing ankle pump exercises and adhere to the physician’s recommendations for fluid replacement to prevent blood concentration (18, 22, 29). Regarding mechanical prevention, IPC serves as the primary method for preventing DVT in patients with cerebral hemorrhage. Nurses must be proficient in the usage and protocols of IPC, while nursing managers should enhance quality control measures within nursing practices. In clinical settings, low patient compliance is a common challenge. Therefore, nurses should prioritize assessing patients’ adherence to ankle pump exercises and mechanical prevention strategies, while also understanding their needs and feelings, and intensifying health education efforts for both patients and their families (40). Nursing documentation plays a crucial role in standardizing the administrative processes undertaken by nurses. These documents serve as formal legal records of nursing services and can provide valuable data for quality improvement and research endeavors (41). Consequently, during the DVT prevention process, nurses must emphasize the importance of accurately recording nursing documentation to ensure standardization in this practice (29).

### 4.5 Limitations and prospects

This study has several limitations. Firstly, while it comprehensively summarizes the best evidence for the prevention of DVT in patients with cerebral hemorrhage, the majority of the literature included originates from other countries. Consequently, the research findings may be influenced by variations in race, region, and cultural background. Secondly, this study is limited to Chinese and English literature, potentially overlooking high-quality research published in other languages. Future research should focus on local studies within China to develop evidence summaries that are more applicable to clinical scenarios in the country. Additionally, as new evidence continues to emerge, it is essential to regularly update this evidence and promote its effective application in clinical practice to enhance the quality of nursing.

## 5 Conclusion

This study employed evidence-based methods to summarize and present 38 key pieces of evidence for the prevention of DVT in patients with cerebral hemorrhage, focusing on six aspects: risk assessment, basic prevention, mechanical prevention, pharmacological prevention, documentation of nursing practices, and informed consent. This evidence summary serves as a scientific and practical resource for clinical medical staff, facilitating the standardization of nursing practices, reducing the incidence of DVT among patients, and providing a foundation for future scientific research initiatives. Furthermore, when implementing this evidence, medical staff should thoroughly consider factors such as the differences in medical conditions both domestically and internationally, as well as the personal preferences of patients, to ensure the careful selection and application of evidence in clinical practice.

## Data Availability

The original contributions presented in the study are included in the article/supplementary material, further inquiries can be directed to the corresponding author.
