# Supplementary material for: Summary of the best evidence for the prevention of deep vein thrombosis in patients with intracerebral hemorrhage
Source: Front Neurol. 2026 Jan 27;17:1752010. doi: 10.3389/fneur.2026.1752010 (PMC12888206; doi:10.3389/fneur.2026.1752010)
Supplement: Supplementary file 1 [file Table_1.DOCX]

**2.1 Problem establishment**

The evidence-based question development tool proposed by the Evidence-Based Nursing Center of Fudan University was utilized [1], and the question was structured according to the PIPOST principle established by the JBI Evidence-Based Health Care Center. The target population for the application of evidence comprises patients with cerebral hemorrhage who are admitted to the neurosurgery ICU. The intervention measures refer to a comprehensive series of strategies aimed at the prevention of DVT, which include basic preventive measures, mechanical prevention, and pharmacological interventions. The professionals responsible for implementing the evidence include nurses and physicians working in the neurosurgical ICU. The outcomes of interest encompass the incidence of DVT among patients as well as the specific locations where DVT occurs. The setting for the application of this evidence is the neurosurgery ICU. The types of evidence considered include relevant clinical practice guidelines, evidence summaries, expert consensus statements, systematic reviews, meta-analyses, clinical decision-making tools, recommended practices, and best practice information resources.

**2.2 Evidence retrieval**

According to the evidence from the '5S' pyramid model [2], a systematic search was conducted across various databases, including UpToDate, BMJ Best Practice, the International Guidelines Collaboration Network, the American Guidelines Network, the National Institute for Health and Clinical Excellence (NICE) of the United Kingdom, and the Joanna Briggs Institute (JBI) Evidence-Based Health Care Database. Additional resources such as the Cochrane Library, Medlink, PubMed, Embase, Web of Clinical Decisions, as well as guidelines, best practices, expert consensus, systematic reviews, and evidence summaries related to Science, CINAHL, Wanfang Database, Chinese Biomedical Literature Database, and CNKI were also included. To ensure the relevance of the evidence, the search period was defined from January 2011 to April 2025.

Search is conducted by combining subject terms and free terms. The Chinese search terms are "deep vein thrombosis, venous thrombosis, deep vein thrombosis", "cerebral hemorrhage, cerebral hemorrhage, parenchymal hemorrhage, intracerebral hemorrhage, cerebral hemorrhage, neurocritical illness", "prevention", and "clinical decision, guideline, clinical practice, expert consensus, systematic review, evidence summary". Take CBM as an example. The search formula is: (Deep vein thrombosis OR venous thrombosis OR deep vein thrombosis) AND (prevention)) AND cerebral hemorrhage OR cerebral hemorrhage OR parenchymal hemorrhage OR intracerebral hemorrhage OR cerebral hemorrhage) AND (clinical decision-making OR guidelines OR best practices OR expert consensus OR systematic review OR evidence summary). With "deep venous thrombos*, venous thrombosis, deep-vein Thrombosis", "pre exposure prophylaxis", Cerebral Hemorrhage, Intracerebral Hemorrhage*, Cerebrum Hemorrhage*, Cerebral Brain Hemorrhage*, Cerebral Parenchymal "Hemorrhage*", "clinical decision, guideline, best practice, systematic review, expert consensu, Summary of evidence" are the English search terms. Take Pubmed as an example. The search formula is: ("venous thrombosis"[MeSH Terms] OR "deep venous thrombos*"[Title/Abstract] OR "Deep-Vein Thrombosis"[Title/Abstract]) AND ("pre exposure prophylaxis"[MeSH Terms] AND ("Cerebral Hemorrhage"[MeSH Terms] OR "Intracerebral Hemorrhage*"[Title/Abstract] OR "Cerebrum Hemorrhage*"[Title/Abstract] OR "Cerebral Brain Hemorrhage*"[Title/Abstract] OR "Cerebral Parenchymal Hemorrhage*"[Title/Abstract]) AND ("clinical decision"[Title/Abstract] OR "guideline"[Title/Abstract] OR "best practice"[Title/Abstract] OR "systematic review"[Title/Abstract] OR "expert consensus"[Title/Abstract] OR "Summary of evidence"[Title/Abstract])

**2.3 Inclusion and exclusion criteria of evidence**

Inclusion criteria for this study encompass patients diagnosed with cerebral hemorrhage or those admitted to the neurosurgery intensive care unit (ICU). The research focuses on intervention measures aimed at preventing DVT, addressing various aspects such as basic preventive strategies, pharmacological interventions, and mechanical prophylaxis. The types of evidence considered include clinical guidelines, expert consensus statements, systematic reviews, evidence summaries, clinical decision-making tools, and best practices pertaining to DVT prevention in patients with cerebral hemorrhage. Only documents published in Chinese or English and possessing a quality evaluation rating above grade C are included. Conversely, the exclusion criteria comprise literature with incomplete data, previously published studies containing identical content, updated guidelines or systematic reviews, and literature for which the full text is unavailable.

**2.4 Literature screening**

After importing the relevant literature into EndNote, duplicate entries were removed. This literature was independently screened by two master's students trained in evidence-based practices. The titles, abstracts, and keywords of the literature were initially pre-screened based on the established inclusion and exclusion criteria. Subsequently, a full-text review was conducted to finalize the selection of included literature.

**2.5 Quality evaluation of the literature**

The included guidelines were evaluated using the Appraisal of Guidelines for Research and Evaluation (2017 Edition) (AGREE II) [3], which encompasses six domains and twenty-three items. Each item is scored on a scale of 1 to 7, with higher scores indicating better guideline quality. The basis for guideline recommendations is as follows: if the standardized percentages across all six domains exceed 60%, it is classified as a strong recommendation (Grade A). If the standardized percentages for three or more domains fall between 30% and 60%, it is classified as a recommendation (Grade B). Otherwise, it is categorized as not recommended (Grade C).

Expert consensus is evaluated using the JBI literature quality assessment tool for expert opinions and professional consensus. This tool comprises six assessment items, each with four response options: "Yes", "No", "Unclear", and "Not applicable" [4]. Systematic review literature was assessed using the evaluation criteria from the Australian JBI Evidence-Based Health Care Centre Systematic Review (2016 Edition) [5]. This tool consists of 11 items, with each item offering four options: "Yes", "No", "Unclear", and "Not applicable".

Given that clinical decision-making, best practices, and evidence summarization are integral to the thematic evidence aggregation process within the evidence-based 5S pyramid, the evidence development process follows a similar structure. Consequently, the CASE (Critical Assessment for Summaries of Evidence) list was utilized to evaluate the literature quality concerning clinical decisions, best practices, and evidence summaries [6]. This tool includes 10 items, each with three response options: "Yes", "No", and "Partially Yes". The literature was independently evaluated by three researchers who had undergone evidence-based training and possessed the necessary qualifications. In cases of disagreement, a fourth researcher was consulted to make the final decision regarding the inclusion of the literature.

**2.6 The extraction, summary of evidence and the determination of evidence levels**

Two researchers summarized the included evidence and extracted it by topic. The extracted content primarily encompasses the subject of the evidence, its description, the source, and its origin. In the event of any conflicts during this process, a third researcher will adjudicate. The principles of evidence aggregation are as follows: if the recommended content is consistent, evidence that is concise and easy to understand will be selected; if the recommended contents are complementary, the evidence will be merged according to logical language flow; and if the recommended content conflicts, the original recommended literature will be revisited to explore the underlying reasons [6]. For the extracted evidence, the 2014 version of the JBI Evidence Pre-Grading and Evidence Recommendation Levels System (JBI Levels of Evidence) [7] was utilized to classify the evidence into grades, which are divided into levels 1 to 5, with Level 1 being the highest and Level 5 the lowest.

Additionally, an expert group meeting was convened, inviting nine multidisciplinary experts to evaluate the evidence based on its FAME attributes: validity, feasibility, applicability, and clinical significance[6]. The strength of evidence is determined using the recommendation levels in the JBI Evidence Pre-Classification and Evidence Recommendation Level System (2014 Edition) and is categorized into strong recommendation (Level A) and weak recommendation (Level B) [8]. The specific evaluation criteria are presented in **Table 1**.

Table 1 Evidence Recommendation Levels of JBI 2014 Edition

| Recommendation level | Judgment criteria |
| --- | --- |
| Grade A recommendation: Strong recommendation | 1. Clearly demonstrate that the intervention measures are more beneficial than detrimental or more detrimental than beneficial.  2. High-quality evidence supports the application.  3. It is beneficial or has no impact on resource allocation.  4. The patient's values, wishes and experiences were taken into consideration. |
| Grade B recommendation:  Weak recommendation | 1. Intervention measures have more advantages than disadvantages or more disadvantages than advantages, although the evidence is not yet clear enough.   2. There is evidence to support the application, although the quality of the evidence is not high enough.  3. It is beneficial to resource allocation, or has no impact, or has a minor impact.  4. Partially consider, or not consider, the patient's values, wishes and experiences. |

**References**

1. Zhu Z,Hu Y,Xing WJ,Zhou YF,Gu Y: The composition of different types of evidence-based problems. J Nurses Train 2017, 32(21):1991-1994.(in Chinese).
2. Alper BS, Haynes RB: EBHC pyramid 5.0 for accessing preappraised evidence and guidance. Evid Based Med 2016, 21(4):123-125.
3. Brouwers MC: The AGREE Reporting Checklist: a tool to improve reporting of clinical practice guidelines (vol 352, i1152, 2016). Bmj-Brit Med J 2016, 354.
4. Wang AH, Yang J, Jiang LL, Chen J, Ma Y, Wang YH: Best evidence summary for aspiration prevention and management in critically ill patients with nasogastric feeding. J Clin Nurs 2025, 34(4):1170-1186.
5. Gu Y,Zhang HW,Zhou YF,Hu Y,Xing WJ: JBI evidence-based health center's quality assessment tool for different types of research—the quality evaluation of diagnostic and economic evaluation. J Nurses Train 2018, 33(8):701-703.(in Chinese).
6. Xie WG, Zhang C, Liu XY, Shu Y, Yang XC, Deng YL: Summary of the best evidence for prevention of deep vein thrombosis in patients with aneurysmal subarachnoid hemorrhage. Chin J Emerg Crit Care Nurs 2024, 5(01):85–91.(in Chinese).
7. Shu Y,Bi MM,Zhang C,Gong YY,Li ZX, Zhou TT, Li XR,Liu XY, Xie WG: Summary of the best evidence of artificial airway endotracheal tube cuff management in ICUadult patients. Chin J Nurs 2022, 57(24):3038-3045.(in Chinese).
8. Wang CQ,Hu Y: JBI evidence pre-classification and evidence rank system (2014 edition). J Nurses Train 2015, 30 (11):964-967.(in Chinese).
